# Noise as a risk factor in the delivery room: A clinical study

**DOI:** 10.1371/journal.pone.0221860

**Published:** 2019-08-30

**Authors:** Kristiane Roed Jensen, Lone Hvidman, Ole Kierkegaard, Henrik Gliese, Tanja Manser, Niels Uldbjerg, Lise Brogaard

**Affiliations:** 1 Department of Obstetrics and Gynecology, HEH-Horsens Regional Hospital, Horsens, Denmark; 2 Department of Obstetrics and Gynecology, Aarhus University Hospital, Aarhus, Denmark; 3 ArbejdsmiljøEksperten A/S, Occupational and environmental consultants, Esbjerg, Denmark; 4 School of Applied Psychology FHNW, University of Applied Sciences and Arts Northwestern Switzerland, Olten, Switzerland; Centre Hospitalier Departementai Vendee, FRANCE

## Abstract

**Introduction:**

We aimed to investigate whether noise in delivery rooms is associated with impaired performance of obstetric teams managing major (≥1000 mL) postpartum hemorrhage.

**Material and methods:**

We included video recordings of 96 obstetric teams managing real-life major postpartum hemorrhage. Exposure was noise defined as the occurrence of sound level pressures (SPL) above 90 dB. The outcome was high clinical performance assessed through expert ratings using the TeamOBS-PPH tool.

**Results:**

The 23 teams unexposed to noise had a significantly higher chance of high clinical performance than the 73 teams exposed to noise: 91.3% (95% CI; 72.0–98.9) versus 58.9% (95% CI; 46.8–70.3) (*p* < 0.001). The results remained significant when adjusting for the following possible confounders: team size, non-technical performance, bleeding velocity, hospital type, etiology of bleeding, event duration and time of day. Typical sources of noise above 90 dB SPL were mother or baby crying, dropping of instruments, and slamming of cupboard doors.

**Conclusion:**

Noise in delivery rooms may be an independent source of impaired clinical performance.

## Introduction

Over the past decades, noise has increased at hospitals throughout the world[1]. The estimated increase is 15 dB(A), which is considerable as an increase of 10 dB corresponds to a doubling of the perceived noise level. Noise is associated with adverse health outcomes, stress and impaired performance[2,3]. Especially sudden, unpredictable noise can cause a startled response and thus lead to work disruption[4–6].

We know little about how noise affects healthcare providers managing acute events in the hospital setting. However, studies point at increased levels of stress and impaired performance with cognitive tasks and motor coordination for healthcare workers and other professionals being affected[7–11]. Accordingly, a pediatric surgery department tried to implement a noise reduction program[12]. They managed to reduce the overall operation theatre sound levels from 63dB(A) to 60 dB(A) and observed a significant decrease in complication rates during the same period of time. Labor wards are even noisier with average noise levels reaching 87 dB(A) and maximum noise levels reaching 122 dB(A)[13]. Even though this is not experienced by all personnel every day, these levels are actually within the range where hearing protection is recommended by the WHO[14]. Such high noise levels may be critical when managing emergencies like postpartum hemorrhage where providing the correct treatment promptly is of paramount importance[15].

If noise has the potential to impair the clinical performance of obstetric teams, precautions should be taken. To our knowledge, no study of the impact of noise on the clinical performance of real-life obstetric emergency teams has previously been published. We therefore aimed to investigate whether exposure to noise impairs clinical performance in obstetric teams managing real-life major (≥1000 mL)[16] postpartum hemorrhage.

## Material and methods

### Study design and setting

In this clinical study, we performed a secondary analysis of soundtracks and video recordings of teams managing real-life major postpartum hemorrhage. As previously described[17], a chip in the obstetrician’s telephone activated the recorders when she or he entered any delivery room at one of two Danish hospitals: a university hospital and a regional hospital. The University Hospital in Aarhus had approximately 5,000 deliveries; provided maternal care at level 3; and was staffed by 20 technicians, 100 midwives and 50 physicians. The Regional Hospital in Horsens had approximately 2,000 deliveries; provided maternal care at level 2; and was staffed by 45 midwives, 2 technicians and 25 physicians. The recorders were installed and inclusion of cases began 20 October 2014 at the Regional Hospital in Horsens and on 10 January 2015 at the University Hospital in Aarhus and ended at both locations on 1 May 2016.

### Noise analysis

The labor ward is a noisy environment, and average noise levels have been estimated at 87 dB(A)[13]. We therefore defined noise exposure as the presence of a sound level pressure above 90 dB and we used that to categorize the teams as being either *exposed* or *never exposed* to noise. To explore the effects of noise level, we also estimated the impact on performance of sound levels up to 90 dB SPL, 85 dB SPL 80 dB SPL, 75 dB SPL and 70 dB SPL.

Sound level was monitored by a microphone (ECM-10/WS, Monacor, Germany) placed in the ceiling right above the patient’s bed and was saved as an audio file (WAV file). The placement of the microphone and the gain settings were the same in all rooms. We analyzed the audio files using the software Praat (computer software, Institute of Phonetic Sciences—University of Amsterdam)[18] which calculated the sound pressure level (SPL) in decibels relative to a reference level of 0.00002 Pa, the threshold of human hearing being 1 kHz. We calculated one measurement of dB SPL every 10.67ms, as recommended by Praat[19], and thereby also calculated the duration (milliseconds) of sound levels. Our method of estimating dB SPL was tested for precision as recommended by the sound engineer (HG) by comparing our results to a Sound Level Meter (Model 2250, Brüel & Kjær, Denmark) in a white noise test-setup at the following distances from the microphone: 1 cm and 1 m. The precision of our estimate is within one dB SPL.

### Performance assessment based on video recordings

The audio and video files of obstetric teams managing real-life patients with major postpartum hemorrhage were assessed for clinical performance and non-technical performance.

*Clinical performance*: Two consulting obstetricians independently assessed all video recordings using the validated TeamOBS-PPH tool[17]. The average score of the two raters was used for the final analysis. The TeamOBS-PPH score (range 0–100) is a measure of how well teams provide patient care according to the protocol. The minimal pass level is 60; below this score, there is a risk of harming the patient. The level associated with high clinical performance is 85, as above this score no or only minor errors affect the score. Inter-rater agreement was (intra class correlation) ICC 0.84;(95%CI; 0.76–0.89).

*Non-technical performance*: Two physicians, independently assessed the video recordings using the “*Assessment of obstetric team performance*” AOTP tool[20] with interrater agreement ICC 0.97; 95%CI; 0.96–0.98). The tool allows assessment of 18 observable non-technical skills in six categories: 1) Communication with the patient and partner; 2) Task management, 3) Teamwork and leadership; 4) Situation awareness; 5) Team communication; 6) Environment of the room (S1 Table). The last category (Environment of the room) specifically covers certain behaviors of team members undertaken to manage distractions such as malfunctioning or missing equipment.

All four raters assessed all 96 videos independently and were blinded to each other’s scores.

Additional information regarding team size, bleeding etiology, time of day for the event, event duration and bleeding amount was collected. At both hospitals, standard procedure prescribes that bleeding amount is estimated by weight and continuously stated vocally by the nurse to the team. Bleeding velocity was estimated using the first and second bleeding amount registered (mL bleeding per minute).

### Statistical analysis

We did not conduct a power calculation because this study is a secondary analysis[21–23]. The chance and the risk difference of high *clinical performance* and noise were estimated with 95% confidence interval (CI) using binary regression analysis[24]. The assumption of the no effect measure modification of noise by team size (number of healthcare providers in the team), non-technical performance (AOTP summative score, score-squared as continuous variable), bleeding velocity (mL bleeding per minute), hospital type (university or regional), etiology (atony, retained placenta, or lacerations) and time of day (time and time-squared as continuous variable, hours) was assessed by including an interaction between the adjusted measure and noise in the binary regression model.

The association between the chance of high clinical performance and the duration of noise exposure in milliseconds was visualized by the nonparametric analysis Lowess[25,26]. STATA 15, Texas was used for all statistical analyses.

### Ethical approval

All participants appearing in the video recordings gave written consent for the videos to be analyzed for research purposes. The study was approved in May 2014 by the Central Denmark Region, the Danish Data Protection Agency (2012-58-006) and the Research Foundation of Central Denmark (case-no. 1-16-02-257-14).

## Results

**Fig 1:**
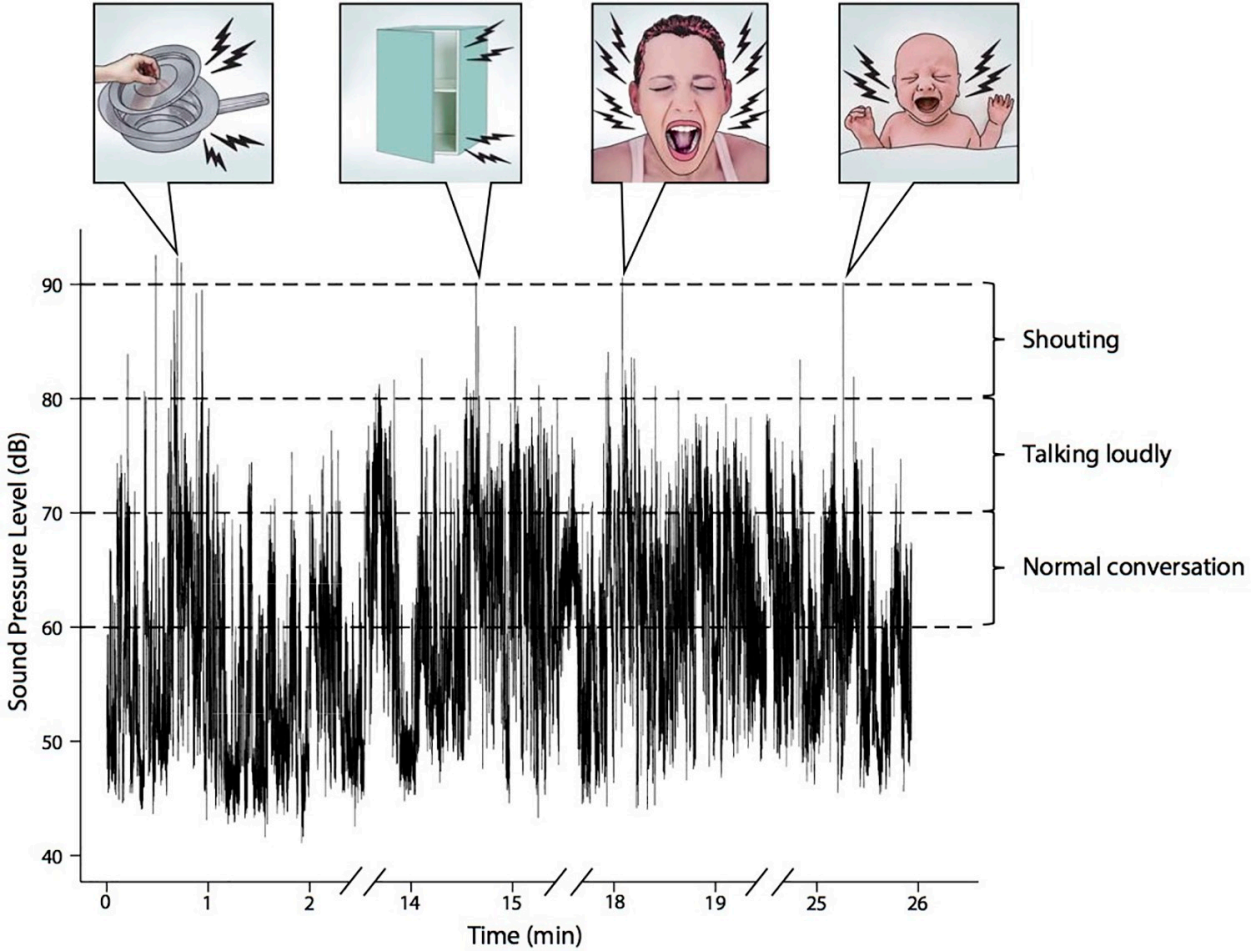
Example of noise level during a postpartum haemorrhage.

In this study we included a total of 99 teams. These teams consisted of 213 different healthcare providers in 97 different combinations. Totaling 60 physicians, 125 midwifes and 28 technicians. After excluding three recordings due to technical error, we analyzed the data associated with 96 obstetric teams managing major postpartum hemorrhage. We found that 73 teams (76%) were exposed to noise defined as a SPL above 90 dB, whereas 23 teams (24%) were never exposed to noise. We identified the sources of noise in the first 50% of the audio files by viewing the audio and video for the time of the noise peak. Typical sources of noise were cupboard doors slamming, metal instruments being dropped and the mother crying loudly (Fig 1). Other sources were chairs being dragged over the floor, the baby crying loudly, coughing and many people talking loudly at the same time.

The teams who had not been exposed to noise had a 91.3% (95%CI; 72.0–98.9) chance of high clinical performance, whereas this chance was only 58.9% (95%CI; 46.8–70.3) for exposed teams (Table 1). This reduction of 32 percentage points (*p* < 0.001) remained significant when adjusting for the following potential confounders separately: team size, non-technical performance, bleeding velocity, hospital type, etiology, event duration, and time of day (Table 2).

**Table 1:** Noise and clinical performance.

Exposed to noise >90dB	High clinical performance*		
	n	Risk %	(95%CI)
No	23	91.3	(72.0–98.9)
Yes	73	58.9**	(46.8–70.3)

*TeamOBS-PPH score ≥85

** p value <0.001

**Table 2:** Possible confounders.

	Difference in risk of high clinical performance Exposed vs. unexposed to noise ≥90 dB			
	n	Risk difference %	(95%CI)	p value
Unadjusted	96	32.4	(16.3–48.5)	<0.001
Adjusted for team size	96	34.2	(20.3–48.2)	<0.001
Adjusted for non-technical performance	96	26.6	(11.1–42.0)	<0.001
Adjusted for bleeding velocity*	76	27.2	(9.1–45.3)	0.003
Adjusted for hospital	96	31.4	(14.9–47.8)	<0.001
Adjusted for etiology	96	31.4	(14.9–47.9)	<0.001
Adjusted for event duration	96	34.8	(23.7–45.9)	<0.001
Adjusted for time of day	96	33.3	(20.5–46.1)	<0.001

*Calculation from first to second measurement. Twenty teams are missing since they only have one measurement during the event.

**Fig 2:**
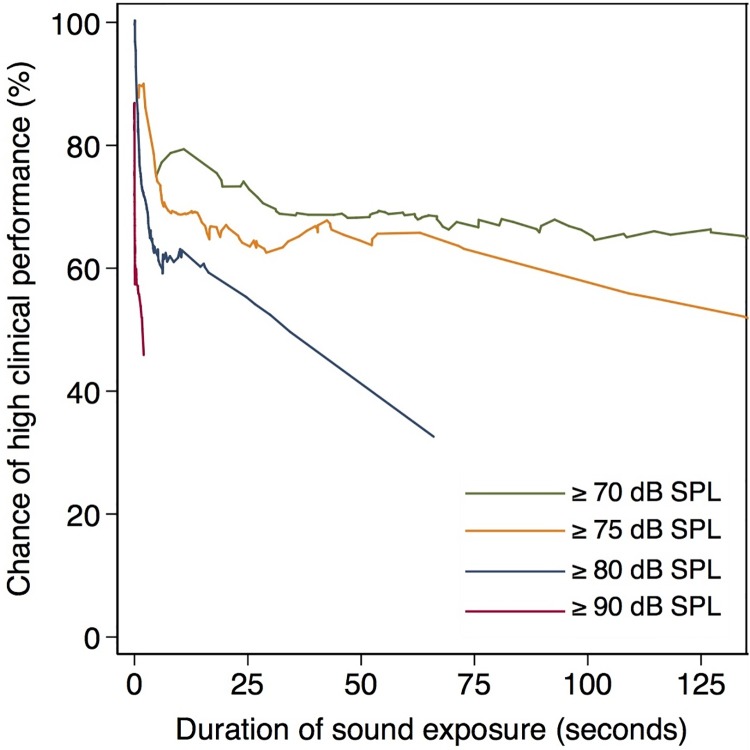
Impact of sound duration on the chance of high clinical performance.

The chance of high clinical performance decreased with the duration of noise (Fig 2). The duration needed to impact performance was about 0.15 seconds at 90 dB SPL, 20 seconds at 80 dB SPL and 90 seconds at 75 dB SPL. No obvious decrease in the chance of high clinical performance was observed for exposure to sound at 70 dB SPL. Further analysis of performance and sound levels are available in the supplemental material, illustrating that the mean dB SPL for the entire recording was negatively associated with the chance of high clinical performance (S1 File).

## Discussion

In this study, we found that teams who had not been exposed to noise above 90 dB SPL had a 91.3% chance of high clinical performance, whereas this figure was only 58.9% for the noise-exposed teams. Typical sources of high noise levels were cupboard doors slamming, metal instruments being dropped or the mother or baby crying.

The main strength of this study is that we were able to collect audio and video data of teams managing real-life obstetric emergencies. Furthermore, our method allowed us to make very exact measurements on fluctuating noise levels as noise was measured every 10.67 millisecond, and the precision of our measurements was within 1 dB SPL. Another strength is the number of different team members. With a total of 60 physicians and 125 midwives participating in the study we achieved high staff heterogeneity thus minimizing the risk of confounding due to personal characteristics.

There were also a number of limitations. 1) We measured the noise using one microphone in the ceiling and therefore do not have an exact measure of the noise exposure for each team member. However, the position of that one microphone was well-chosen as it was placed above the foot of the patient’s bed and thus reflected the noise exposure experienced by those leading the team. 2) The sample did not allow us to adjust for a combination of all confounders in a multivariate analysis; however, none of the individual confounders seemed to affect the results (Table 2 and S1 Table). 3) We defined noise as SPL > 90 dB, which make out results comparable to those from other studies [27] even though the choice of this cutoff resulted in an unbalanced division of the population into 73 exposed teams and 23 non-exposed teams. 4) Furthermore, by our definition, a very short peak of noise categorized the team as noise exposed. One could argue that the average noise level during the entire video recording is also relevant. We therefore addressed this secondary finding in S1 File, which shows a similar association between noise and clinical performance.

This study cannot establish causality between noise and performance. However, we find it likely that noise impacts on team performance in the delivery ward. Our results were unaffected by performance-relevant confounders: team size, non-technical performance, bleeding velocity, hospital type, etiology, event duration, and time of day. Nevertheless, there remains a risk of unmeasured confounding. In particular, a number of other possible distractors related to the procedure such as doors opening/closing, equipment missing, case-irrelevant conversations, and self-initiated interruptions[28–30]. Distractions and interruptions during clinical activities have been investigated in anesthetic teams and associated with a negative impact on the patient management[31]. These distractors have not been accounted for in the present study. Future research with adequate power should therefore consider a wider range of distractors that may affect team performance in the delivery room.

While our findings indicate a significant effect of noise on performance, it is noteworthy that there are noise-exposed teams with high clinical performance. Explanations could be that some people are more susceptible to noise[3,32], that some sources of noise are more disruptive and that performance is also affected by other factors. Further studies are needed to assess these aspects in the future.

We find it likely that noise impacts on team performance; however, one could also argue the opposite that staff become more noisy when the clinical situations deteriorate, i.e. a poor clinical performance induces a tense situation and stresses the staff and the mother and baby. If so, our conclusion concerning the influence of noise on the clinical performance is wrong; in fact, the direction might be reversed. We acknowledge this hypothesis. However, based on existing literature[2,3], we find our original hypothesis more likely as noise was not associated with bleeding velocity in the delivery room.

In the present study, we estimated sound level pressure using a non-weighted decibel scale. A number of studies have used an A-weighted decibel scale, as the purpose of A-weighting is to compensate for our perception’s dependence on sound frequency[33]. However, A-weighting is not ideal for measuring high noise levels (above 60 dB), and it has a tendency to underestimate the level of low-frequency noise[34].

We defined noise as sound level above 90 dB SPL, because the average sound level during labor has been estimated to be 87 dB(A)[13]. However, moderate sound levels above 80 dB SPL and 75 dB SPL but not 70 dB SPL also affected team performance (Fig 2), albeit at more than 100-fold longer durations. This finding supports results from previous studies where moderate noise did not affect performance as negatively as high noise[35–38]. One must remember that a single 90 dB SPL noise peak does not necessarily represent the whole sound to which the team is exposed.

This disparity between the teams’ experience of noise and our measurements of noise peaks is visualized in Fig 1, where a neonate cries for several minutes, but this only results in a single noise peak above 90dB.

Noise reduction may improve the teams’ working environment and thereby their clinical performance. Several initiatives may be considered to reduce the exposure to noise: pain relief before bimanual palpation, soft closing system on cabin doors and a warning system to the delivery ward, e.g. using a SoundEar^™^ to increase the teams’ awareness of noise. Though wall-to-wall carpeting and heavy curtains are not an option due to strict hygiene regulations, noise can be reduced in a simple manner by placing mineral wool insulation behind the ceiling[39].

## Conclusion

In conclusion, our study suggests that high noise levels reduce clinical performance in obstetric emergency teams. To prove causality, randomised controlled trials of noise reduction are needed. However, on the basis of our findings, we recommend that noise reduction be considered to support optimal performance and patient care during obstetric emergencies.

## Supporting information

S1 Table Description of items in AOTP. (PDF)

S1 File Supplemental analysis. (DOCX)

## Abbreviations

AOTP: Assessment of obstetric team performance
dB SPL: Sound Pressure Level in decibel
dB(A): A-weighted sound level
dB: Decibel
