# Supplementary material for: Noise as a risk factor in the delivery room: A clinical study
Source: PLoS One. 2019 Aug 30;14(8):e0221860. doi: 10.1371/journal.pone.0221860 (PMC6716652; doi:10.1371/journal.pone.0221860)
Supplement: S1 Table — (PDF) [file pone.0221860.s001.pdf]

S1 Table: Description of items in AOTP

Category/Item	Excellent team performance
**1. Communication with patient**	
1.1 Information sharing	*Introduction and role identification by team members as they enter the room; address patient/partner directly; team continues to update patient.*
1.2 Reassuring attitude	*At least one team member assumes the responsibility to continuously reassure the patient/partner.*
1.3 Partner management	*Team anticipates disruptive behavior and intervenes early to prevent escalation.*
**2. Task/Case management**	
2.1 Plan of action	*Explicit urgency of situation identified; differential diagnosis developed and plan of action shared with team members; patient well prepared for procedures; team member's verify readiness to proceed with interventions.*
2.2 Resources utilization	*People and equipment used effectively; efficient method of acquiring resources.*
2.3 Problem solving	*Team adapts to situation; timely recognition of problem; continuous attempt to resolve problem.*
**3. Teamwork**	
3.1 Leadership	*Leader identified; constructive style of leadership that encourages participation and team success; leader engages team members for improvement.*
3.2 Role assignment	*Roles and responsibilities of team members clear, quickly establish roles.*
3.3 Team interaction	*Trust and respect demonstrated by all team members; flattened hierarchy encourages contribution of opinions; team members monitor and provide feedback on each other's performance; team members openly accept help.*

**4. Situation awareness**	
4.1 Anticipation	*Early recognition of impending problem; preemptive actions taken (e.g. call for blood, call for extra help).*
4.2 Realizing limitations	*Team members acknowledge need for help; early call for help; extra personnel summoned in a timely fashion; early recognition of limitations related to knowledge; skills and ability.*
4.3 Avoiding fixation	*Consider the global situation.*
4.4 Responsiveness	*Rapid response to critical situation.*
4.5 Vigilance	*Team members alert to clinical situation.*
**5. Team communication**	
5.1 Focused communication	*Clear questions/requests for action directed to a specific person identified by name; instructions given in clear voice which can be easily heard; orderly communication.*
5.2 Closing the loop	*Sender / receiver acknowledges that message has been received; team members repeat what is being asked of them to ensure that correct actions are taking place; sender requests confirmation when actions are complete; confirmation provided when requested actions are completed.*
**6. Environment of the room**	
6.1 Management of disruption	*Dialogue focused on clinical situation; no conversations about personal issues; disruptive behaviors (e.g. yelling, rudeness to team members) dealt with efficiently to regain focus on clinical situation.*
6.2 Atmosphere in the room	*Voices remain calm allowing focus on care; calm with voice and body language; controlled demeanor; orderly and controlled environment.*

Note. Descriptions of excellent performance are from the Handbook of Assessment of Obstetric Team performance.[20]
