# Supplementary material for: Noise as a risk factor in the delivery room: A clinical study
Source: PLoS One. 2019 Aug 30;14(8):e0221860. doi: 10.1371/journal.pone.0221860 (PMC6716652; doi:10.1371/journal.pone.0221860)
Supplement: S1 File — (DOCX) [file pone.0221860.s002.docx]

**Supplemental analysis**

The main research question was to evaluate the association between exposure to noise and the chance of high clinical performance. This analysis was conducted using a binary regression analysis with clinical performance as a binary outcome and noise as a binary explanatory variable.

However, both clinical performance and noise can also be analyzed as continuous variables.

Clinical performance was assessed by the tool TeamOBS-PPH resulting in a score from 0-100. In the binary analysis, we dichotomized the score into high (score >85) and low performance. We analyzed clinical performance as a continuous score using multiple linear regression, with noise as a binary variable, assessing the mean difference in clinical performance score between teams exposed or non-exposed to noise (Table S1). The unadjusted analysis and the adjusted analysis for all potential confounders were very similar. The strength of multiple linear regression analysis is the possibility to adjust for the complete set of the potential confounders as confidence intervals and statistical tests are exact, assuming that the model is correct. The limitation of the linear regression analysis is that the mean difference in score does not clarify whether it affects low or high performing teams.

| **S2 Table Clinical performance and effects of potential confounders** | | | | |
| --- | --- | --- | --- | --- |
|  | **Mean difference in clinical performance score**  Exposed vs. unexposed to noise ≥90 dB | | | |
|  | **n** | **Score**** | **(95%CI)** | **p value** |
| Unadjusted | 96 | 5.7 | (0.95 - 10.4) | 0.019 |
| Adjusted for team size | 96 | 5.8 | (1.1 - 10.6) | 0.016 |
| Adjusted for bleeding velocity* | 96 | 4.2 | (-0.83 - 9.3) | 0.100 |
| Adjusted for hospital | 96 | 5.6 | (0.94 – 10.4) | 0.003 |
| Adjusted for etiology | 96 | 5.5 | (0.76 – 10.2) | 0.023 |
| Adjusted for event duration | 96 | 5.8 | (1.06 – 10.6) | 0.017 |
| Adjusted for time of day | 96 | 2.3 | (0.85 - 10.4) | 0.021 |
| Adjusted for all confounders above | 96 | 5.2 | (0.19 – 10.2) | 0.042 |
| *Calculation from first to second measurement. Twenty teams are missing since they only have one measurement during the event.  ** Clinical performance score from two raters using TeamOBS-PPH | | | | |

**S1 Fig Chance of high clinical performance and mean sound pressure level (dB)**

Noise was assessed as sound pressure level in decibel. In the binary regression analysis, we dichotomized sound pressure level into noise (>90 dB) and no noise. To assess noise as a continuous variable in the original decibel scale, we estimated the chance of high clinical performance as a function of mean sound pressure level using a Lowess curve (Figure S2). The strength of this analysis is the possibility to visualize the importance of mean sound level pressure and the limitation is that the mean dB does not clarify whether team members have been exposed to noise (>90 dB).
